# Beta Diversity Patterns and Drivers of Macroinvertebrate Communities in Major Rivers of Ningxia, China

**DOI:** 10.3390/ani15142034

**Published:** 2025-07-10

**Authors:** Qiangqiang Yang, Zeyu Wei, Xiaocong Qiu, Zengfeng Zhao

**Affiliations:** 1 School of Civil and Hydraulic Engineering, Ningxia University, Yinchuan 750021, China; qqy1995@126.com (Q.Y.); weizy02619@163.com (Z.W.); zhaozf0517@163.com (Z.Z.); 2 School of Life Sciences, Ningxia University, Yinchuan 750021, China

**Keywords:** macroinvertebrates, beta diversity, optimal parameter-based geographical detector (OPGD), generalized dissimilarity modelling (GDM), normalized stochasticity ratio (NST)

## Abstract

The clarification of community assembly mechanisms in benthic macroinvertebrates and their respective contributions to the development of beta diversity is a fundamental concern in aquatic ecology. Nonetheless, the intrinsic complexity of community alterations and their non-linear reactions to gradients of explanatory variables present considerable obstacles to measuring the determinants of beta diversity. Fifty sampling points were set up along the major rivers of the Yellow River Irrigation Area (YRIA), the Central Arid Zone (CAZ), and the Southern Mountainous Area (SMA) in Ningxia in April, July, and October 2023. The findings demonstrate that the optimal parameter-based geographical detector (OPGD) model identified a 3000 m circular buffer as the spatial scale at which landscape structure most significantly influences water quality. A degradation in water quality presumably results in diminished differences in species composition among communities. The Sørensen index was determined to be more appropriate for this investigation, and the total beta diversity of the communities was relatively high (β_SOR_ ≥ 0.82), with no identifiable nested spatial patterns detected. Except in the YRIA, environmental variability contributed more significantly to the variance in beta diversity than spatial factors, and deterministic mechanisms dominated the community assembly of benthic macroinvertebrates across all three months. To improve biodiversity and aquatic ecosystem health, the study region should optimize its landscape structure by reducing the amount of bare land and increasing the percentage of forest land within buffer zones. Additionally, a multi-site conservation strategy should be put into place.

## Simple Summary

Elucidating the community assembly mechanisms of macroinvertebrates and their relative contributions to the formation of beta diversity is a central issue in aquatic ecology. In this study, we employed Generalized Dissimilarity Modelling to quantify the relative importance of explanatory variables in shaping community beta diversity. We further identified the dominant assembly processes by integrating Generalized Dissimilarity Modelling with the Normalized Stochasticity Ratio and determined the most appropriate beta diversity metric for the study region. The findings provide a scientific basis for optimizing land use configurations and guiding aquatic biodiversity assessment and conservation.

## 1. Introduction

Water, which has many different uses and functions that are vital to the sustainable provision of human well-being, is universally acknowledged as the most critical natural resource [1]. However, readily accessible freshwater sources—such as wetlands, rivers, and lakes—constitute less than 1% of the Earth’s total water volume [2]. Among the most frequently used freshwater resources, rivers are unique and irreplaceable ecosystems whose openness, dynamism, and longitudinal connection make them crucial for ecological integrity and societal advancement [3,4,5]. Urbanization, agriculture, and dam construction are examples of human activities that change the spatial arrangement of vegetation and habitats in watersheds, causing habitat fragmentation and deterioration of water quality [6]. These changes exacerbate biotic homogenization and decrease aquatic biodiversity [7,8].

Beta diversity can be viewed as being based on the equilibrium between alpha diversity and gamma diversity [9], offering a robust framework for evaluating biotic homogeneity [10]. Deterministic processes (e.g., environmental filtering, whereby species are sorted along environmental gradients and occupy their optimal habitats) and stochastic processes (e.g., dispersal limitation) jointly govern beta diversity during community assembly [11,12], with their relative contributions varying across spatial and temporal scales [13]. Community assembly mechanisms establish the ecological foundation for the emergence of beta diversity, which subsequently reflects the spatiotemporal results of these mechanisms [14]. Environmental filtering, which favours the preservation of adapted species, and allopatric speciation, resulting from geographic isolation, can improve beta diversity [15]. Collinearity, however, affects the measurement of the independent effects of these two ecological processes [16]. Prior research has primarily utilized techniques including the Mantel test (or partial Mantel test), linear matrix regression, and multiple regression on dissimilarity matrices to investigate the relationship between beta diversity and environmental gradients [17,18,19]. Nonetheless, the non-linear correlation among these variables diminishes the precision of the outcomes obtained from these techniques [20]. Variance partitioning analysis (VPA) has been employed to investigate the distinct impacts of several variables (or groups of variables) on the development of beta diversity [21]. Nevertheless, the quantity of groups is frequently restricted, failing to address the common effect problem resulting from factor collinearity [22]. Effectively quantifying the relative contributions of ecological processes to forming beta diversity is a key issue in maintaining biodiversity [23].

Macroinvertebrates are characterized by wide geographic distributions, high species richness, and limited dispersal capabilities [24]. Owing to their sensitivity to environmental fluctuations, they are regarded as sentinels [25]. They provide several ecological functions, including enhancing organic detritus decomposition, facilitating nutrient flows, and regulating food webs [26,27]. Consequently, they are regarded as optimal species for examining the mechanisms that govern the development of beta diversity [28].

The Ningxia Hui Autonomous Region (Ningxia) has advanced irrigation-based agriculture to foster regional economic development, but this has also intensified non-point source pollution, such as nitrogen pollution, and heightened ecological risks to aquatic organisms [29,30]. This study centres on macroinvertebrates, aiming to (1) ascertain the patterns of beta diversity within macroinvertebrate communities, (2) elucidate the applicability of two types of beta diversity calculation schemes within the study area, and (3) uncover the predominant roles of stochastic and deterministic processes in community assembly.

## 2. Materials and Methods

### 2.1. Study Area

**Figure 1:**
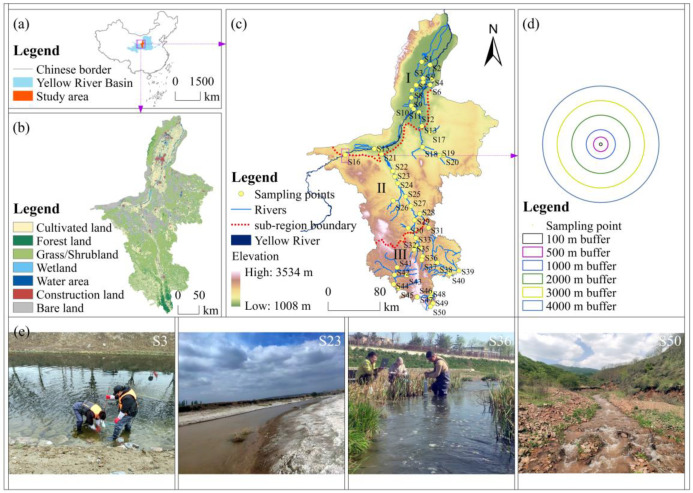
(**a**) Geographic position of Ningxia in Northwest China; (**b**) land use classifications in 2020; (**c**) spatial distribution of sub-regions and sampling points in Ningxia; Regions (I–III) correspond to YRIA, CAZ, and SMA, respectively; (**d**) six categories of circular buffer zones; (**e**) representative photos of some sampling points.

Ningxia (35°14′~39°23′ N, 104°17′~107°39′ E) encompasses an area of 66,400 km^2^ and is the sole provincial-level territory in China that is situated entirely inside the Yellow River Basin. The area has a moderate continental arid and semi-arid climate, with an average annual temperature of 5 °C to 9 °C and annual precipitation of 150 mm to 600 mm [31]. The region can be categorized into three zones from north to south, based on multi-year average precipitation lines (200 mm and 400 mm) and other environmental attributes, including agricultural and pastoral distributions: the Yellow River Irrigation Area (YRIA), the Central Arid Zone (CAZ), and the Southern Mountainous Area (SMA) [32,33]. Our study monitored macroinvertebrates and physicochemical factors across these three zones in April, July, and October 2023. The quantity of monitoring sites in YRIA, CAZ, and SMA was 16 (S1–S16), 14 (S17–S30), and 20 (S31–S50), respectively (Figure 1).

### 2.2. Collection of Datasets

#### 2.2.1. Benthic Macroinvertebrates

Beginning at the upstream segment of the monitoring transect, macroinvertebrate collection was performed over a 100 m river reach using a D-net (mesh size: 0.5 mm; frame dimensions: 30 cm × 30 cm), with six replicate samples collected at each point from different habitats and pooled into a single composite sample [34]. Samples were filtered using a 40-mesh screen, packaged in polyethylene zip-lock bags, and transferred to the laboratory under refrigeration. Upon arrival, they were arranged on white porcelain trays for sorting, fixed in 75% ethanol, and stored in 50 mL specimen vials. Identification and enumeration were primarily conducted using a dissecting microscope (LIOO SMZ61, Beijing, China), and biological abundance was calculated as the number of individuals per square metre (ind./m^2^). Identification of macroinvertebrates relied predominantly on the aquatic invertebrate reference literature [35,36], with specimens classified according to the most specific taxonomic unit achievable.

#### 2.2.2. Environmental Factors

At each sampling site, the latitude, longitude, and altitude (ALT) were recorded in the field. Simultaneously, physicochemical parameters—including river width (RW), pH, water temperature (WT), dissolved oxygen (DO), electrical conductivity (EC), flow velocity (VEL), turbidity (TUR), chlorophyll *a* (Chl *a*), salinity (SAL), total dissolved solids (TDS), and fluoride (F^−^)—were measured. Water was collected using a 1 L sampler at 0.5 m below the surface, with three parallel samples combined into a single composite. In reaches shallower than 0.5 m, sampling was performed at mid-depth. The composite sample was used to rinse 500 mL polyethylene bottles prior to filling; the bottles were then sealed, stored at 0~4 °C in a refrigerator, and transported to the laboratory for analysis at the earliest opportunity. The levels of pollutants, including ammonia nitrogen (NH_3_-N), total nitrogen (TN), total phosphorus (TP), nitrate (NO_3_^−^-N), nitrite (NO_2_^−^-N), and the permanganate index (COD_Mn_), were assessed in accordance with the national water quality standards HJ 535-2009 (Water quality—Determination of ammonia nitrogen—Nessler’s reagent spectrophotometry), HJ 636-2012 (Water quality—Determination of total nitrogen—Alkaline potassium persulfate digestion UV spectrophotometric method), GB/T 11893-1989 (Water quality—Determination of total phosphorus—Ammonium molybdate spectrophotometric method), GB 7480-87 (Water quality—Determination of nitrate-Spectrophotometric method with phenol disulfonic acid), GB 7493-87 (Water quality—Determination of nitrogen (nitrite)—Spectrophotometric method), and GB 11892-89 (Water quality—Determination of benzene and its analogies—Gas chromatographic method).

#### 2.2.3. Land Use

Liu et al. [37] created a 10 m resolution land-cover map for China in 2020, utilizing a deep classification network and Sentinel-2 imagery as data sources. The projected overall accuracy was 84.35% ± 0.92%. This dataset exhibits enhanced classification accuracy and spatial detail compared to other land-cover products (e.g., GlobeLand30, ESA WorldCover, Esri Land Cover, and FROM-GLC-10), rendering it particularly suitable for the requirements of this study. According to the current land use classification (GB/T 21010-2017) and the specific conditions of the study region, the land was classified into seven categories: cultivated land, forest land, grass/shrubland, construction land, water area, wetland, and bare land.

### 2.3. Methods

#### 2.3.1. Environmental Heterogeneity

The geographical distance between sampling points was computed using the distm function from the “geosphere” package [38] in R (v.4.3.3), utilizing their latitude and longitude coordinates. The vertical distance between two points was calculated using the Euclidean distance approach through the dist function. Environmental dissimilarity (Ed) between sampling locations was determined based on environmental variables (Equation (1)) [39], and environmental heterogeneity was assessed based on environmental dissimilarity.

(1) $$\mathrm{Ed}=\left(\mathrm{Euc}/{\mathrm{Euc}}_{\max}\right)+0.001$$

where Euc signifies the Euclidean distance between two sample points, while Euc_max_ indicates the maximum Euclidean distance within the distance matrix. The inclusion of 0.001 is mainly designed to address the null similarity between sampling points.

#### 2.3.2. Beta Diversity Measurement

The pairwise Sørensen dissimilarity index was employed to compute the beta diversity (β_sor_) between sample points, utilizing binary species presence/absence data. According to the additive partitioning approach introduced by Baselga [40], β_sor_ can be divided into species turnover (β_sim_) and species nestedness (β_nes_). The multiple-site Sørensen index was utilized to compute the overall beta diversity (β_SOR_), which was subsequently divided into turnover (β_SIM_) and nestedness (β_SNE_) to evaluate the contributions of various components. Simultaneously, utilizing species abundance data, the beta.pair.abund function was employed to assess the Bray–Curtis dissimilarity index (d_BC_) between locations, which was subsequently partitioned into the balanced variation component (d_BC-bal_) and the abundance gradient component (d_BC-gra_), with d_BC-bal_ and d_BC-gra_ corresponding to β_sim_ and β_nes_, respectively [40]. The beta.multi.abund function was employed to calculate the Bray–Curtis index (β_BC_) for measuring overall dissimilarity, which was then divided into the balanced variation component (β_BC_._BAL_) and the abundance gradient component (β_BC_._GRA_) [41]. Beta diversity was calculated primarily using the R package “betapart”.

#### 2.3.3. Buffer Zone Screening

Considering the retention and bi-directionality of the rivers, as well as the multidirectional water sources, circular buffer zones (100 m, 500 m, 1000 m, 2000 m, 3000 m, and 4000 m) centred around sampling points were established using ArcGIS 10.8, following approaches from previous studies [42]. Concurrently, the “GD” package was utilized to determine the optimal parameter-based geographical detector (OPGD) model, particularly in the parameter optimization segment, to ascertain the critical spatial scales affecting water quality [43]. The *q* value (*q* ∈ [0, 1]) signifies the explanatory capacity of independent variables, with elevated values indicating more explanatory strength. The ideal scale is established when the 90th percentile of all independent variables attains its peak value. The influencing factors were classified into three categories: (1) the distribution of various land use types within the buffer zones; (2) landscape metrics at the landscape level, comprising the Largest Patch Index (LPI), Contagion Index (CONTAG), and Shannon’s Diversity Index (SHDI), with their ecological significance detailed in Table S1; (3) the human activity intensity of land surface (*HAILS*) (Equations (S1) and (S2)) [44].

The water quality index (*WQI*; Equation (S3)) integrates multiple physicochemical parameters into a single weighted value that reflects the overall status of the aquatic environment [45,46,47,48,49]. Thirteen indicators—pH, WT, EC, TUR, DO, TDS, TN, NH_3_-N, NO_2_^−^-N, NO_3_^−^-N, TP, COD_Mn_, and Chl *a*—were included in the *WQI* calculation. Details of the parameter settings, calculation procedure, and water quality classification scheme are provided in the Supplementary Material. The mean *WQI* for April, July, and October was used as the response variable.

### 2.4. Statistical Analysis

Shapiro–Wilk’s test and Levene’s test demonstrated that the *WQI* values for different months and different zones within the same month both followed a normal distribution (*p* > 0.05) and exhibited homogeneity of variance (*p* > 0.05). Therefore, independent-sample *t*-tests were employed to compare water quality among sections within the same month, while linear mixed-effects models (LMMs) were used to assess differences across months. *p*-values were adjusted using the Bonferroni correction. LMM analyses were conducted primarily with the R packages “lme4”, “lmerTest”, and “emmeans”.

Generalized Dissimilarity Modelling (GDM) analysis was conducted utilizing the “gdm” package, employing the gdm function and formatsitepair function to investigate the underlying mechanisms of beta diversity [18]. This analysis utilized the beta diversity matrix across sampling sites as the response variable, with geographic distance, altitude distance, and environmental heterogeneity as predictor variables. The number of I-splines and the positions of the “knots” were established at their default values. The partial response plots of the fitted predictor factors demonstrate that the peak value on the *y*-axis signifies the extent of the variable’s contribution to community variation, whereas the curve’s slope represents the rate of change in the response variable along the predictor variable’s gradient [50].

The significance of stochastic and deterministic processes in macroinvertebrate community assembly was evaluated using the Normalized Stochasticity Ratio (NST) [51]. The NST was predominantly computed utilizing the tNST function from the “NST” package, employing the “bray” distance matrix and the “PF” null model [52].

## 3. Results

### 3.1. Composition of Species

**Figure 2:**
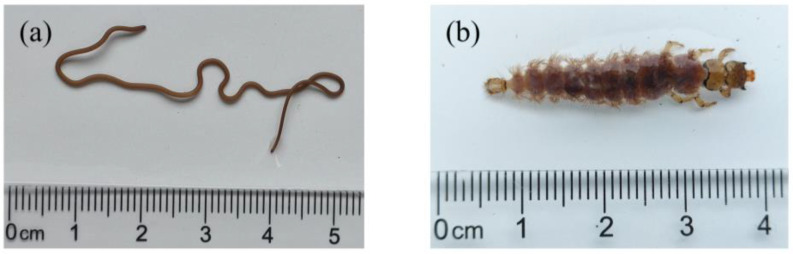
(**a**) *Gordius aquaticus*; (**b**) *Himalopsyche* sp.

Sixty-nine taxonomic units of macroinvertebrates were found, classified into 4 phyla, 7 classes, 20 orders, and 58 families (Figure S1). Arthropoda constituted the majority, with 72.5% of the total taxonomic units, followed by Mollusca at 18.8%. In contrast, Nematomorpha was the least represented, with only one species, *Gordius aquaticus*, collected (Figure 2a). A novel record in Ningxia, *Himalopsyche* sp. (Figure 2b), was identified. This species was obtained from a rapidly moving stream in the Liupan Mountains (sampling location S50, Figure 1c, Figure 1e S50). The pronotum is completely sclerotized, whereas the mesonotum and metanotum are wholly membranous. The mesothorax, metathorax, and abdominal segments one to eight possess prominent lateral gills. This species is classed as a predator under the family Rhyacophilidae of the order Trichoptera [53].

### 3.2. Beta Diversity and Its Components

**Figure 3:**
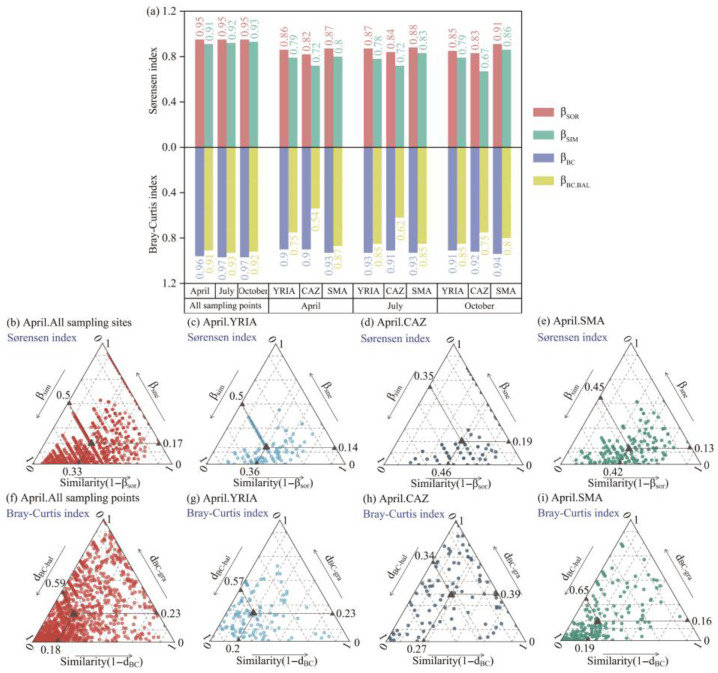
Analysis of macroinvertebrate beta diversity. (**a**) Overall beta diversity and characterization of its components; (**b**–**e**) ternary plots of beta diversity computed with the incidence-based Sørensen index in April; (**f**–**i**) ternary plots of beta diversity computed with the incidence-based Bray–Curtis index in April. Note: Each filled circle represents a pair of sampling sites; the grey triangle denotes the overall mean across all site pairs; and the small triangles along each side illustrate how that mean is decomposed into the respective components.

Analysis utilizing the multiple-site Sørensen index and Bray–Curtis index of dissimilarity indicated that overall beta diversity was notably high across various months or spatial scales (β_SOR_ ≥ 0.82, β_BC_ ≥ 0.9), with β_SIM_/β_SOR_ and β_BC_._BAL_/β_BC_ both above 0.5 (Figure 3a). This suggests that species turnover is a primary factor influencing alterations in beta diversity, with no notable nested pattern detected.

Consistent with the overall beta diversity, the pairwise Sørensen dissimilarity index analysis revealed that the mean species composition similarity (1 − β_sor_) was below 0.5, with β_sim_/β_sor_ above 59%, signifying a greater influence of turnover components (Figure 3b–e, Figures S2a–d and S3a–d). For example, in April, the average beta diversity across 1225 pairs of sampling sites in all sampling points was 0.67, with turnover and nested components accounting for 74.63% and 25.37%, respectively. Compared to the Sørensen index, the mean species composition similarity (1 − d_BC_) derived from the Bray–Curtis index was diminished (Figure 3f–i, Figures S2e–h and S3e–h). Moreover, in the CAZ between April and July, d_BC-gra_ exceeded d_BC-bal_ (Figure 3h and Figure S2g), signifying the predominance of the nested component.

### 3.3. Determinants of Beta Diversity Patterns

#### 3.3.1. Water Quality Evaluation and Optimal Buffer Zone Selection

**Figure 4:**
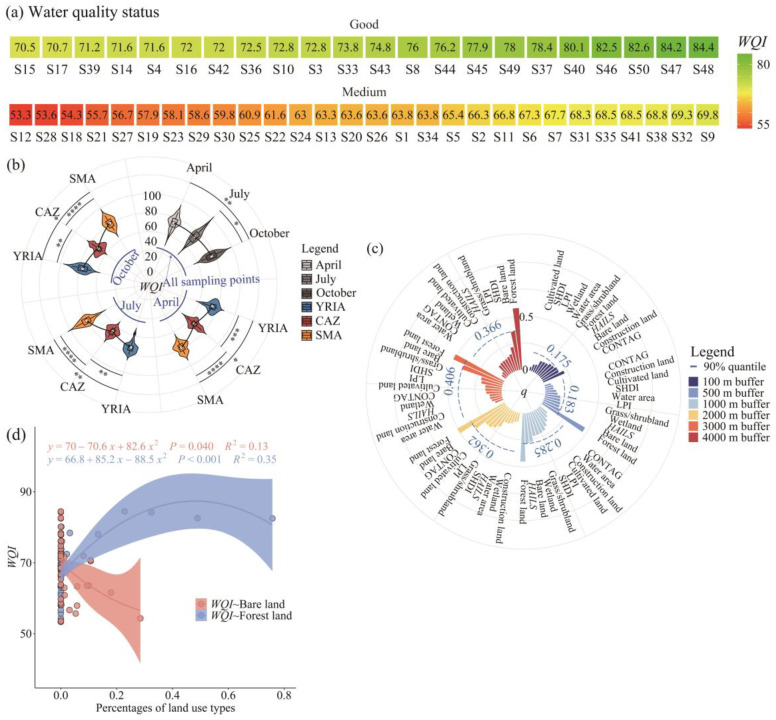
(**a**) Average *WQI* values for April, July, and October; (**b**) analysis of *WQI* variations between months and across distinct zones within the same month; (**c**) results of optimal buffer zone selection derived from the OPGD model; (**d**) correlation of the ratio of forested land to bare land with the *WQI*. Note: *, *p* ≤ 0.05; **, *p* ≤ 0.01; ***, *p* ≤ 0.001; ****, *p* ≤ 0.0001.

The average *WQI* readings for April, July, and October varied from 53.3 to 84.4, classifying water quality into two tiers. Of the sampling sites, 44% were designated as having “Good” water quality, while 56% were categorized as “Medium” (Figure 4a). In October, water quality was optimum, with no significant variations noted between April and July (*p* > 0.05) (Figure 4b). Furthermore, the water quality across the three months exhibited the hierarchy of SMA > YRIA > CAZ (*p* < 0.05), signifying that the SMA had the highest water quality, while the CAZ had the lowest (Figure 4b).

The examination of the OPGD model indicated that the 90th percentile of *q*-values for the 11 driving factors had an early increase, followed by a decline, peaking at 0.406 within the 3000 m buffer zone (Figure 4c). Consequently, the 3000 m buffer zone was chosen for further research. At this spatial scale, forest land (*q* = 0.520) and bare land (*q* = 0.406) were recognized as the principal environmental factors influencing spatial variance in water quality. As the percentage of forest area rose, the *WQI* initially ascended and subsequently declined (*p* < 0.001). In contrast, as the percentage of bare land rose, the *WQI* correspondingly diminished (*p* = 0.04) (Figure 4d).

#### 3.3.2. Analysis of the Factors Influencing Beta Diversity

**Figure 5:**
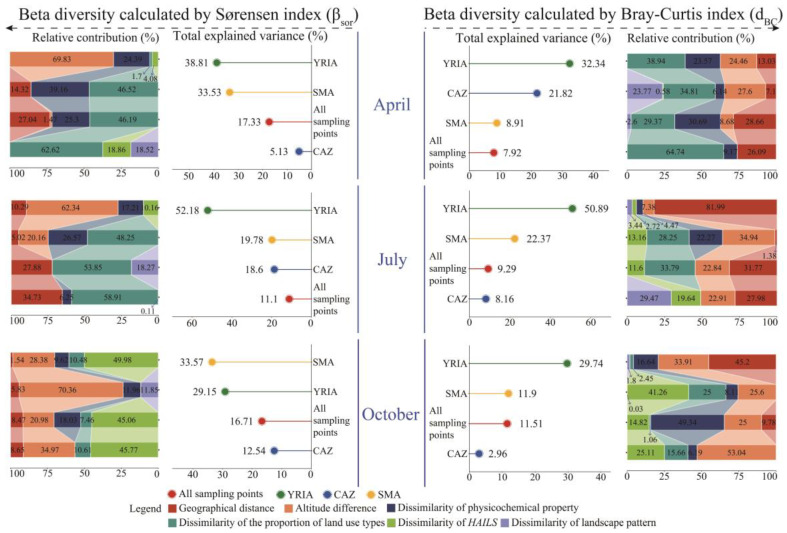
Total explained variance and relative contribution of predictors to beta diversity of macroinvertebrate community.

The GDM fitted six predictor variables to β_sor_ and d_BC_: geographical distance, altitude difference, dissimilarity of physicochemical property, dissimilarity of the proportion of land use types, dissimilarity of *HAILS*, and dissimilarity of landscape pattern (Figure 5). Overall, in the same month and region, the total explained variance of the predictor variables for β_sor_ exceeded that for d_BC_. For example, in April, the total explained variance of the predictor variables for β_sor_ in the YRIA, the SMA, all sampling points, and the CAZ was 38.81%, 33.53%, 17.33%, and 5.13%, respectively; only for the CAZ was β_sor_ lower than d_BC_ (21.82%). The relative contributions of the predictor factors to β_sor_ and d_BC_ exhibited significant variance. Except for the YRIA, the cumulative explained variance of geographical distance and altitude difference for β_sor_ was inferior to that for the other four predictor variables. However, no such trend was noted for d_BC_.

### 3.4. Community Assembly Mechanisms of Macroinvertebrates

**Figure 6:**
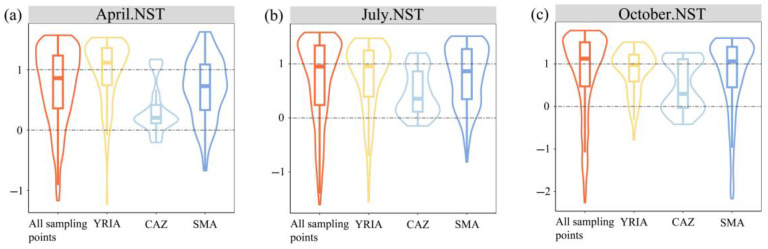

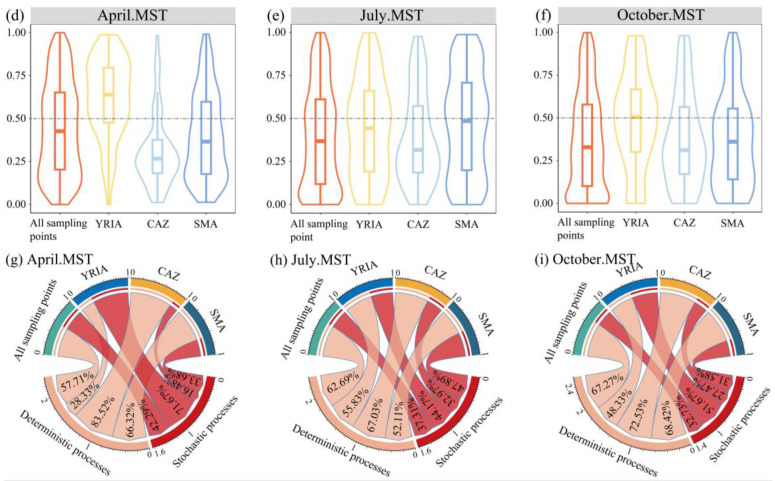
The ecological assembly mechanisms of macroinvertebrate communities. (**a**–**c**) NST values; (**d**–**f**) MST values; (**g**–**i**) the ratio of stochastic to deterministic processes derived from the MST values. MST > 0.5 and MST < 0.5, respectively, indicate the predominance of stochastic and deterministic processes in community assembly.

The NST analysis indicated that the NST values were outside the 0~1 range (Figure 6a–c), necessitating the application of the modified stochasticity ratio (MST) to assess the relative significance of two ecological processes in community assembly. The distribution of MST values is illustrated in Figure 6d–f. At all sampling points, deterministic processes accounted for 57.71% in April, 62.69% in July, and 67.27% in October, whereas the share of stochastic processes was comparatively minor and exhibited a progressive decline. Regionally, the percentage of MST values below 0.5 surpassed the 50% criterion in both the CAZ and the SMA, signifying a more pronounced effect of deterministic selection in both areas, with the CAZ exhibiting a stronger deterministic influence than the SMA. In the YRIA, the percentages of MST values over 0.5 in April and October were 71.67% and 51.67%, respectively, signifying a more pronounced impact of stochastic processes, although the severity of this impact waned over time.

## 4. Discussion

### 4.1. Optimal Selection of Buffer Zones

Scale effects are prevalent in geographical spatial analysis [54]. Landscape composition also exhibits scale dependence in relation to water quality [55]. Establishing appropriate management buffer widths holds regional and temporal importance [56]. Techniques such as redundancy analysis (RDA), stepwise multiple linear regression (SMLR), partial least squares structural equation modelling (PLS-SEM), and VPA are frequently employed to determine the optimal spatial scale of landscape structure’s influence on water quality [57,58,59,60]. Wang et al. [61] emphasized the viability of such methodologies (e.g., RDA) in their analysis. It is worth noting that the response of water quality to environmental gradients is frequently non-linear [62,63], even though these methodologies often presume linear correlations to examine variable linkages [64,65]. This presents constraints in tackling intricate non-linear correlations or collinearity challenges in ecological data [22,66]. Consequently, this paper presents the OPGD model. The Geographical Detector (GD) model operates without assumptions regarding the variables, and can quantify both linear and non-linear connections [67,68]. Moreover, in contrast to the GD model, the OPGD model addresses challenges such as inadequate discretization effects and subjectivity, thereby enhancing the model’s resilience and enabling it to more precisely reveal the magnitude of individual factors’ effects on the dependent variable [69].

The analysis indicated that 3000 m is a breakpoint affecting water quality in the study area, denoting the ideal buffer zone. Huang et al. [70] revealed that landscape configuration within a 5.5 km radius buffer exerted the most substantial influence on water quality. The primary cause of this mismatch may stem from the varied determination methods utilized. Furthermore, the argument persists over which geographical sizes (e.g., circular, riparian, or watershed) have the most substantial impact on water quality [71]. Mello et al. [72] posited that watershed scales have greater influence than riparian zones; however, Zhou et al. [73] asserted that riparian zones have a more pronounced effect than both watershed and circular buffers. The regional heterogeneity of geographical phenomena, the diversity of data sources, and the complex interplay of human and environmental forces result in a lack of consensus on the optimal spatial scale [74,75,76]. Consequently, evaluating local conditions and using context-specific strategies is essential when establishing the ideal spatial scale for river management.

### 4.2. Analysis of Beta Diversity and Its Influencing Mechanisms

Various methodologies exist for quantifying beta diversity; however, a consensus on which method most correctly addresses particular ecological concerns has yet to be established [77]. The Sørensen index, based exclusively on incidence data, is straightforward to comprehend and compute, and has been extensively utilized in beta diversity assessments [78]. Integrating abundance data, the Bray–Curtis index is more resilient to undersampling—a failure to document all species present at a sampling site—than earlier indices [79,80]. This study calculated the beta diversity of macroinvertebrate communities using the Sørensen and Bray–Curtis indices. The outcomes from multiple-site and pairwise dissimilarity analyses reveal substantial discrepancies between the beta diversity estimates derived from the two indices. For instance, the frequency distribution of β_sor_ within the value range of sample pairs in the ternary plot typically has a parabolic shape, with a peak occurring within a specific central range. Conversely, d_BC_ demonstrates the greatest proportion inside the (0.9, 1] range, exhibiting a declining tendency as the interval values diminish (Figures S4–S6). Errors are prevalent in empirical research, rendering the selection of relatively precise methods for evaluating beta diversity more crucial [81].

GDM is a matrix-regression-based approach that is insensitive to multicollinearity among predictor variables and is capable of automatically selecting predictors during model fitting [82]. Furthermore, by incorporating generalized linear modelling with I-spline basis functions, GDM effectively uncovers non-linear relationships in ecological datasets [83]. The GDM method was employed to assess the significance of individual predictors in relation to beta diversity (Figure 5). At the same time, the partial response plots validated the non-linear associations among environmental gradients, geographical factors, and alterations in community composition (Figures S7–S12), aligning with prior research [84,85,86]. Furthermore, these findings suggest that linear models (e.g., Mantel test) are inadequate in capturing and characterizing the dynamic responses of beta diversity to various environmental gradients. Concerning the relevance of the two beta diversity calculation methods in the research area, the pairwise Sørensen dissimilarity index yielded results more compatible with total beta diversity. Secondly, in general, the total explained variance of the predictors for β_sor_ changes exceeded that for d_BC_. Ultimately, the MST analysis indicated that except for the YRIA in April and October, the composition of macroinvertebrate communities was primarily influenced by deterministic processes, suggesting that environmental variables were more significant than spatial factors [87]. A comparison of the analysis results between GDM and the MST revealed that the spatial factors (geographical distance and altitude difference) explained the cumulative variation in β_sor_ with a discrepancy only in July for the YRIA, where the GDM analysis suggested that stochastic processes primarily drove the variation in community composition, whereas the MST indicated that deterministic processes were the dominant driver of community assembly. Both methods showed high consistency for the remaining months and regions. However, the analysis of d_BC_ using GDM exhibited a greater divergence from the MST results. The community assembly mechanism dictates the species composition of various communities [88,89], and variations in species composition represent beta diversity. Accordingly, studying beta diversity using the Sørensen index is more pertinent in this domain.

At the scale of all sampling points, the similarity among communities (1 − β_sor_) was seen to be minimal (Figure 3b, Figures S2a and S3a), signifying a rising trend in beta diversity with the enlargement of the spatial scale. This result aligns with the research conducted by He et al. [90] regarding macroinvertebrates. It is noteworthy that, according to GDM fitting, the predicted variables accounted for 17.33%, 11.1%, and 16.71% of the variance in β_sor_ for April, July, and October, respectively (Figure 5). The considerable amount of unexplained variance indicates the existence of other factors influencing the disparities in species composition across communities. Factors such as precipitation, substrate type, river morphology, and hydrological connectivity [91,92] require further investigation. For the YRIA, the relative contribution of altitude difference to β_sor_ exceeded 62%, and its partial response plot demonstrated that the pace of change in beta diversity escalated with more significant altitude differences (Figures S7–S9). Two possible reasons may elucidate the observed occurrence. Firstly, the study area includes various aquatic habitat types, such as drainage ditches (e.g., S3 and S4), the primary channel of the Yellow River (e.g., S14 and S16), and its tributaries (e.g., S12 and S13). Secondly, prolonged irrigation impacts have resulted in diverse alterations in hydrodynamic conditions at various elevations, with the consequent local microhabitats contributing to ecological filtration [93,94]. Compared to other places, the CAZ demonstrated the most remarkable community resemblance (Figure 3d, Figures S2c and S3c), mainly attributable to the inferior water quality in this area (Figure 4b), which diminished the beta diversity [27].

### 4.3. Limitations and Prospects

This study preliminarily explores the applicability of beta diversity and two indices from a species perspective, offering valuable insights for developing conservation strategies for macroinvertebrates. However, the formation of and variation in beta diversity in macroinvertebrates in rivers is an exceedingly complex process. Focusing solely on the species dimension, while neglecting interspecies functional trait variation (functional diversity) and evolutionary differences (phylogenetic diversity), may impose certain limitations on the analysis of community dynamics and associated ecological processes [95,96,97]. An extensive investigation of benthic macroinvertebrate beta diversity over all three dimensions would enhance comprehension of the mechanisms driving alterations in species composition within communities [98].

In addition to the two aspects of spatial beta diversity and its temporal variation explored in this study, beta diversity also encompasses temporal beta diversity and its spatial variation. Research on temporal beta diversity and its spatial dynamics helps to elucidate the stability of communities in different habitats, highlighting environmental characteristics that influence species fluctuations, thereby enabling the effective conservation of specific sites [99]. Future researchers may investigate beta diversity characteristics from a temporal perspective.

## 5. Conclusions

The OPGD model determines the optimal riparian buffer width for explaining water-quality variability and lays the foundation for identifying the drivers of macroinvertebrate beta diversity. GDM captures non-linear community responses to environmental gradients and spatial factors, quantifying the relative contribution of each predictor. Combined with the MST, it selected the appropriate beta diversity metric for the Ningxia reach and elucidated the underlying assembly mechanisms. By integrating OPGD, GDM, and the MST into a unified framework and incorporating multidimensional beta diversity analyses across spatial and temporal scales, the approach provides local conservation managers with a scientific foundation for delineating spatial priorities and guiding ecological restoration, thereby enhancing the health of riverine ecosystems. Although developed using the Ningxia reach as its study area, the framework is inherently region-neutral in its data requirements (land use layers, routine water quality indicators, and macroinvertebrate survey data), scale settings (modifiable according to data resolution and management objectives), and model assumptions, demonstrating strong potential for application across diverse river systems. Future inter-basin comparative studies will be instrumental in assessing the applicability of this framework under varying climatic and habitat conditions, thereby further enriching and refining riverine ecosystem management strategies.

## Data Availability

Data will be made available on request.

## Supplementary Materials

The following supporting information can be downloaded at https://www.mdpi.com/article/10.3390/ani15142034/s1. Figure S1: Composition of macroinvertebrates in Ningxia; Figure S2: Beta diversity of macroinvertebrates and characteristics of its two components at different sampling scales in July; Figure S3: Beta diversity of macroinvertebrates and characteristics of its two components at different sampling scales in October; Figure S4: Beta diversity and its component decomposition characteristics between paired sampling sites in April; Figure S5: Beta diversity and its component decomposition characteristics between paired sampling sites in July; Figure S6: Beta diversity and its component decomposition characteristics between paired sampling sites in October; Figure S7: Partial response graph of GDM for analyzing βsor in April; Figure S8: Partial response graph of GDM for analyzing βsor in July; Figure S9: Partial response graph of GDM for analyzing βsor in October; Figure S10: Partial response graph of GDM for analyzing dBC in April; Figure S11: Partial response graph of GDM for analyzing dBC in July; Figure S12: Partial response graph of GDM for analyzing dBC in October; Table S1: The landscape pattern index and its significance at the landscape level; Table S2: Conversion coefficients of different land use types for construction land equivalent; Table S3: The relative weights (Pi) and normalized values (Ci) of water quality parameters.
