# Supplementary material for: Beta Diversity Patterns and Drivers of Macroinvertebrate Communities in Major Rivers of Ningxia, China
Source: Animals (Basel). 2025 Jul 10;15(14):2034. doi: 10.3390/ani15142034 (PMC12291867; doi:10.3390/ani15142034)
Supplement: Supplementary file 1 [file animals-15-02034-s001.zip › animals-3637471-supplementary.pdf]

# Beta Diversity Patterns and Drivers of Macroinvertebrate Communities in Major Rivers of Ningxia, China

**Qiangqiang Yang [1], Zeyu Wei [1], Xiaocong Qiu [2,*] and Zengfeng Zhao [1]**

[1] School of Civil and Hydraulic Engineering, Ningxia University, Yinchuan 750021, China
[2] School of Life Sciences, Ningxia University, Yinchuan 750021, China
* Correspondence: qiu_xc@nxu.edu.cn

**Table S1** Landscape pattern index and its significance at the landscape level

| Landscape index | Ecological significance |
|---|---|
| LPI | 0 < LPI ≤ 100, the smaller the value of this index, the more fragmented the landscape is. |
| CONTAG | This index reflects the degree of extension of different patch types in the landscape. 0 < CONTAG ≤ 100, the higher the degree of aggregation of patch types, the greater the value of this index. |
| SHDI | This index reflects landscape heterogeneity. SHDI ≥ 0, SHDI increases as the number of different patch types increases and/or the proportional distribution of area among patch types becomes more equitable. |

Notes: LPI, Largest Patch Index; CONTAG, Contagion Index; SHDI, Shannon's Diversity Index. The above landscape indices were calculated in Fragstats 4.2, and the meaning of each index is referred to its Help Contents.

Human activity intensity of land surface (*HAILS*)

$$HAILS = \frac{S_{CLE}}{S} \times 100\% \quad \text{(S1)}$$

$$S_{CLE} = \sum_{i=1}^{n} \left( SL_i \times CI_i \right) \quad \text{(S2)}$$

where *HAILS* represents the human activity intensity of land surface; $S_{CLE}$ refers to the area of construction land equivalent; $S$ is the total area; $n$ is the number of land use types; $SL_i$ and $CI_i$ represent the area and conversion coefficient of construction land equivalent for

Class I land use type, respectively. $CI_i$ is shown in Table S2 [1].

**Table S2** Conversion coefficients of different land use types for construction land equivalent

| Land use type | Cultivated land | Forest land | Grassland | Construction land | Water area | Wetland | Unused land |
|---|---|---|---|---|---|---|---|
| $CI_i$ | 0.2 | 0 | 0.067 | 1 | 0 | 0 | 0 |

Water quality index

According to the study by Pesce et al. [2], the Water Quality Index (*WQI*) was utilized for water quality assessment. The principal calculating formula is delineated in Equation S3.

$$WQI = \frac{\sum_{i=1}^{n} C_i P_i}{\sum_{i=1}^{n} P_i} \tag{S3}$$

Where $n$ represents the total number of selected water quality parameters; $C_i$ denotes the normalized value of parameter $i$, which is assigned based on the measured concentration with reference to the *Environmental quality standards for surface water* (GB 3838-2002); $P_i$ is the relative weight of parameter $i$, where $P_i \in [1, 4]$. A higher value of $P_i$ indicates greater importance of the corresponding parameter in relation to the preservation of aquatic life or human water use [3]. This study selected 13 physicochemical parameters for calculating the *WQI*, the specific parameters of which are detailed in Table S3 [4–6]. According to the computation results, water quality can be categorized into five classifications (Table S4), with elevated *WQI* values signifying superior water quality[4].

**Table S3** The relative weights ($P_i$) and normalized values ($C_i$) of water quality parameters

| Parameter | Units | $P_i$ | $C_i$ | | | | | | | | | | |
|---|---|---|---|---|---|---|---|---|---|---|---|---|---|
| | | | 100 | 90 | 80 | 70 | 60 | 50 | 40 | 30 | 20 | 10 | 0 |
| pH | NA | 1 | 7 | 7-8 | 7-8.5 | 7-9 | 6.5-7 | 6-9.5 | 5-10 | 4-11 | 3-12 | 2-13 | 1-14 |
| WT | ℃ | 1 | 21/16 | 22/15 | 24/14 | 26/12 | 28/10 | 30/5 | 32/0 | 36/-2 | 40/-4 | 45/-6 | >45/<-6 |
| EC | μS/cm | 1 | <750 | <1000 | <1250 | <1500 | <2000 | <2500 | <3000 | <5000 | <8000 | ≤12,000 | >12,000 |
| TUR | NTU | 2 | <5 | <10 | <15 | <20 | <25 | <30 | <40 | <60 | <80 | ≤100 | >100 |
| DO | mg/L | 4 | ≥7.5 | >7 | >6.5 | >6 | >5 | >4 | >3.5 | >3 | >2 | ≥1 | <1 |
| TN | mg/L | 2 | <0.1 | <0.2 | <0.35 | <0.5 | <0.75 | <1 | <1.25 | <1.5 | <1.75 | ≤2 | >2 |
| $NH_3$-N | mg/L | 3 | <0.01 | <0.05 | <0.1 | <0.2 | <0.3 | <0.4 | <0.5 | <0.75 | <1 | ≤1.25 | >1.25 |
| $NO_2^-$-N | mg/L | 2 | <0.005 | <0.01 | <0.03 | <0.05 | <0.1 | <0.15 | <0.2 | <0.25 | <0.5 | ≤1 | >1 |
| $NO_3^-$-N | mg/L | 2 | <0.5 | <2 | <4 | <6 | <8 | <10 | <15 | <20 | <50 | ≤100 | >100 |
| TP | mg/L | 1 | <0.01 | <0.02 | <0.05 | <0.1 | <0.15 | <0.2 | <0.25 | <0.3 | <0.35 | ≤0.4 | >0.4 |
| $COD_{Mn}$ | mg/L | 3 | <1 | <2 | <3 | <4 | <6 | <8 | <10 | <12 | <14 | ≤15 | >15 |
| TDS | mg/L | 2 | <100 | <500 | <750 | <1000 | <1500 | <2000 | <3000 | <5000 | <10,000 | ≤20,000 | >20,000 |
| Chl a | μg/L | 3 | <1 | <4 | <7 | <10 | <15 | <20 | <30 | <40 | <50 | ≤65 | >65 |

Notes: WT, water temperature; EC, electrical conductivity; TUR, turbidity; DO, dissolved oxygen; TN, total nitrogen; $NH_3$-N, ammonia nitrogen; $NO_2^-$-N, nitrite nitrogen; TP, total phosphorus; TDS, total dissolved solids; Chl *a*, Chlorophyll *a*.

**Table S4** Water Quality Condition Grading Criteria for *WQI* Evaluations

| Indicators | Excellent | Good | Medium | Poor | Very poor |
|---|---|---|---|---|---|
| *WQI* | *WQI* > 90 | 70 < *WQI* ≤ 90 | 50 < *WQI* ≤ 70 | 25 ≤ *WQI* ≤ 50 | *WQI* < 25 |

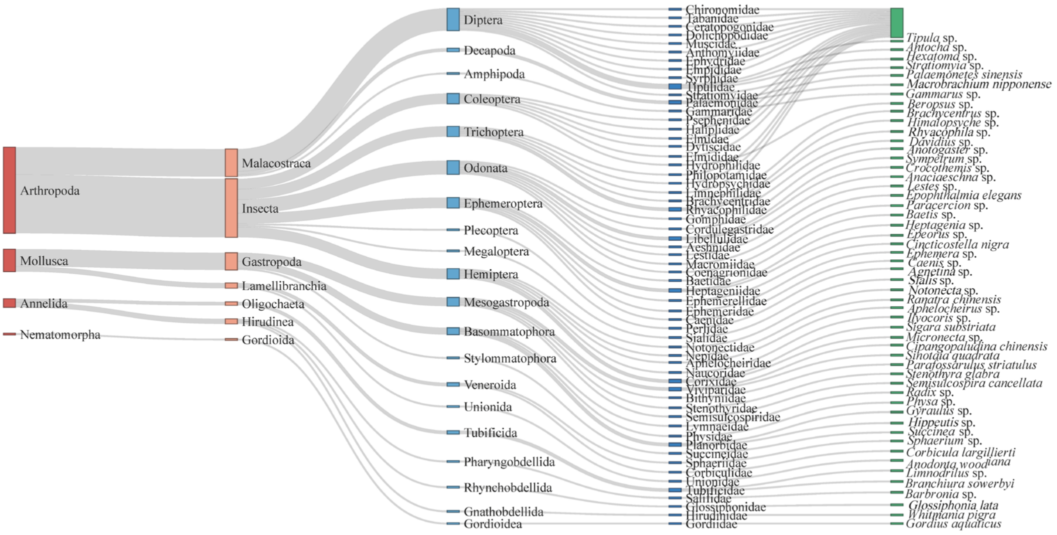


**Figure S1.** Composition of macroinvertebrates in the Ningxia

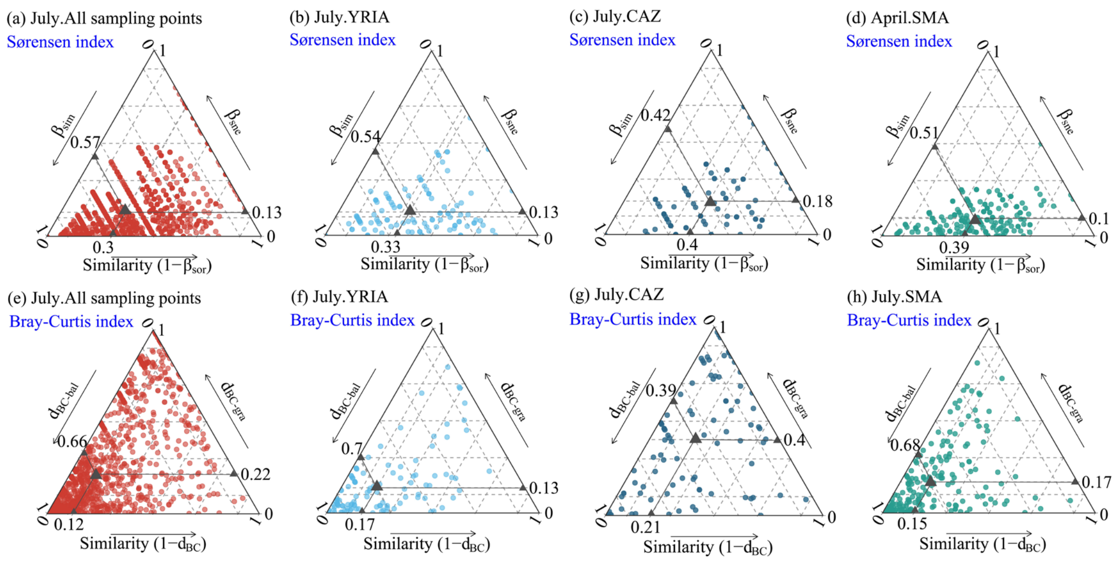


**Figure S2.** Beta diversity of macroinvertebrates and the characteristics of its two components at different sampling scales in July

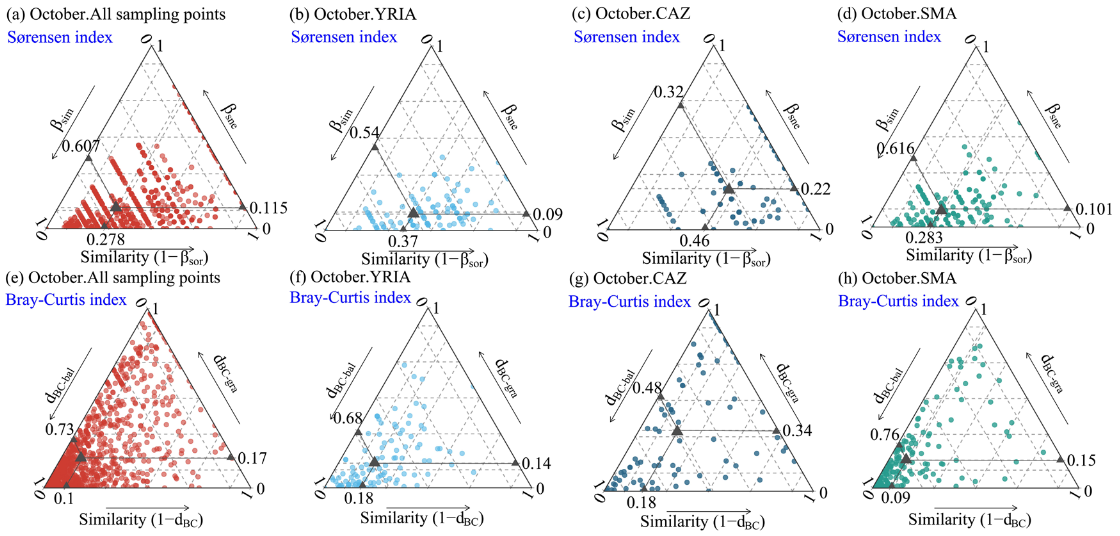


**Figure S3.** Beta diversity of macroinvertebrates and the characteristics of its two components at different sampling scales in October

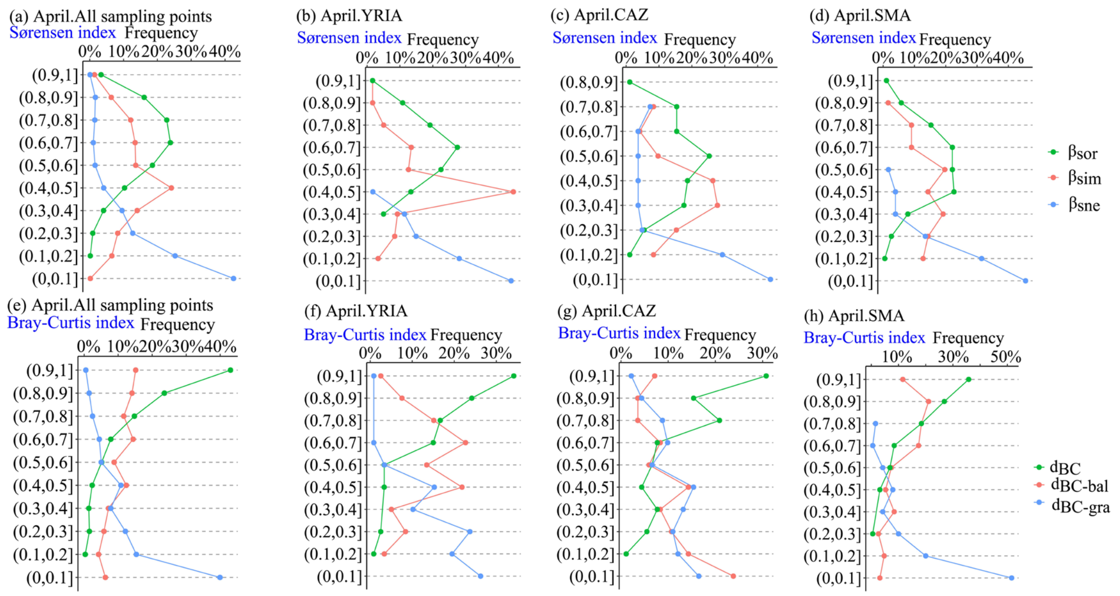

**Figure S4.** Beta diversity and its component decomposition characteristics between paired sampling sites in April

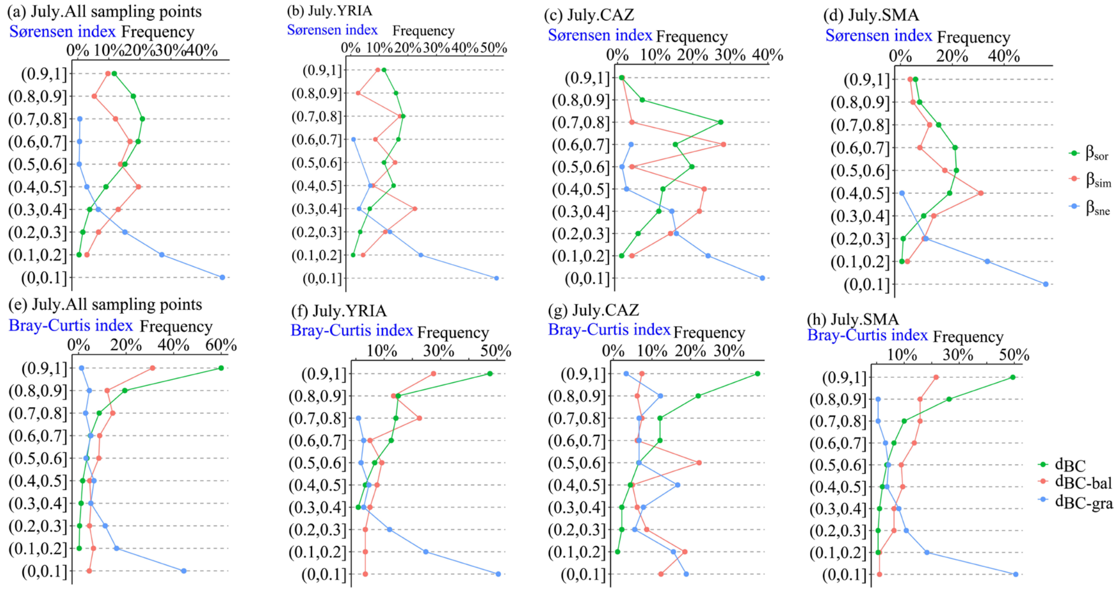

**Figure S5.** Beta diversity and its component decomposition characteristics between paired sampling sites in July

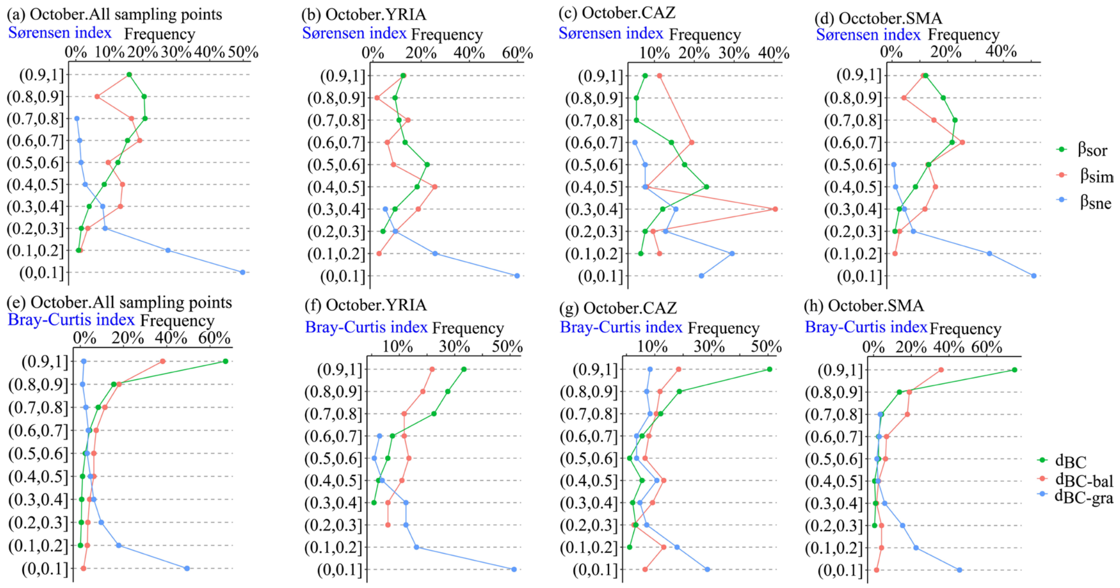


**Figure S6.** Beta diversity and its component decomposition characteristics between paired sampling sites in October

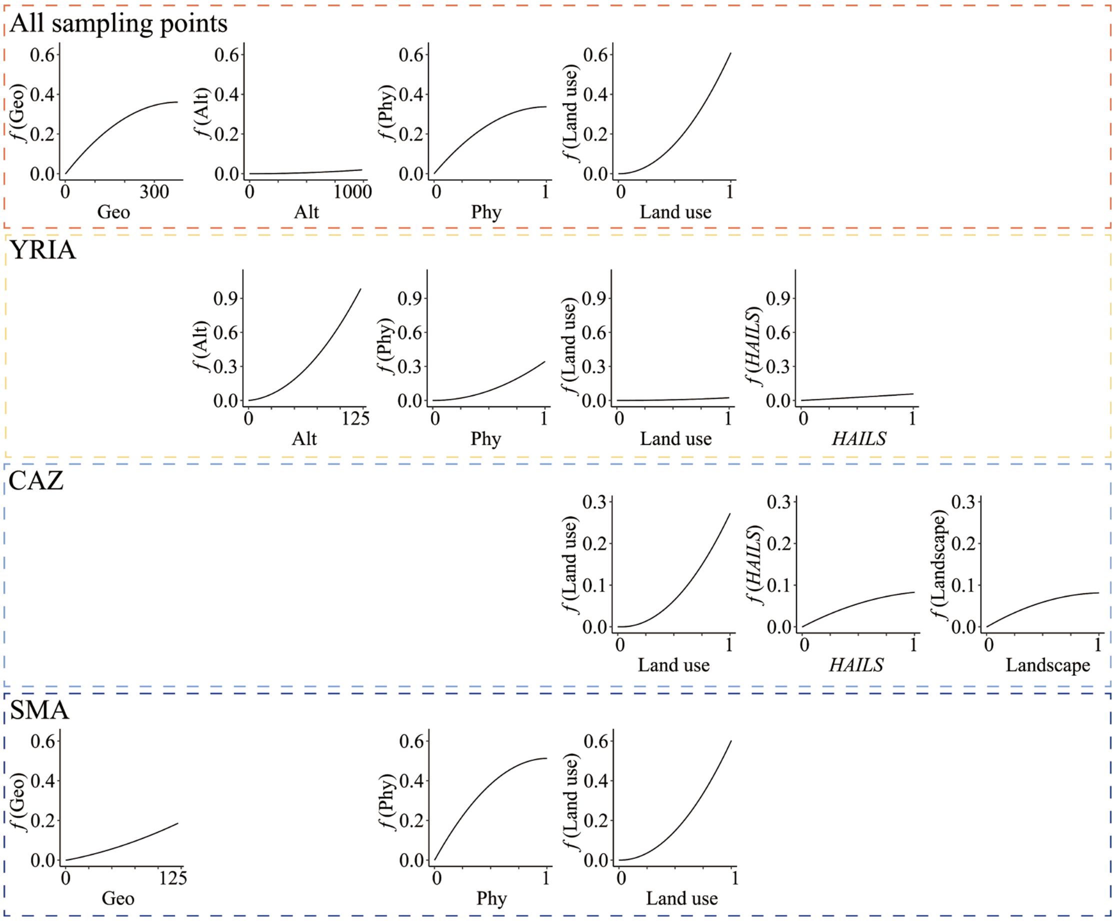


**Figure S7.** Partial response graph of GDM for analyzing $\beta_{sor}$ in April

Note: Geo: Geographical distance; Alt: Altitude difference; Phy: Dissimilarity of physicochemical property; Land use: Dissimilarity of the proportion of land use types; *HAILS*: Dissimilarity of *HAILS*; Landscape: Dissimilarity of landscape pattern. The unit on the x-axis was the original unit for the variable; the function f (variable) on *the y-axis indicated the* I-spline-transformed function.

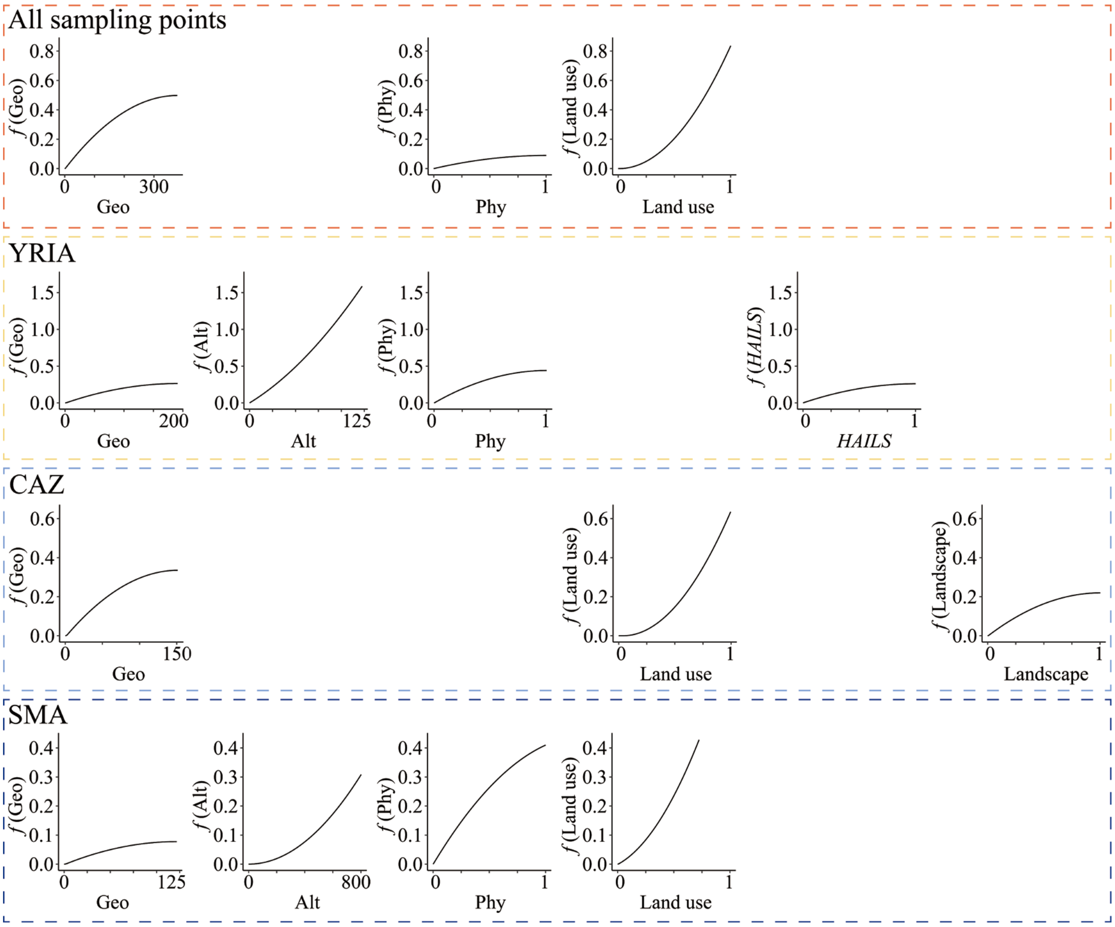


**Figure S8.** Partial response graph of GDM for analyzing $\beta_{sor}$ in July

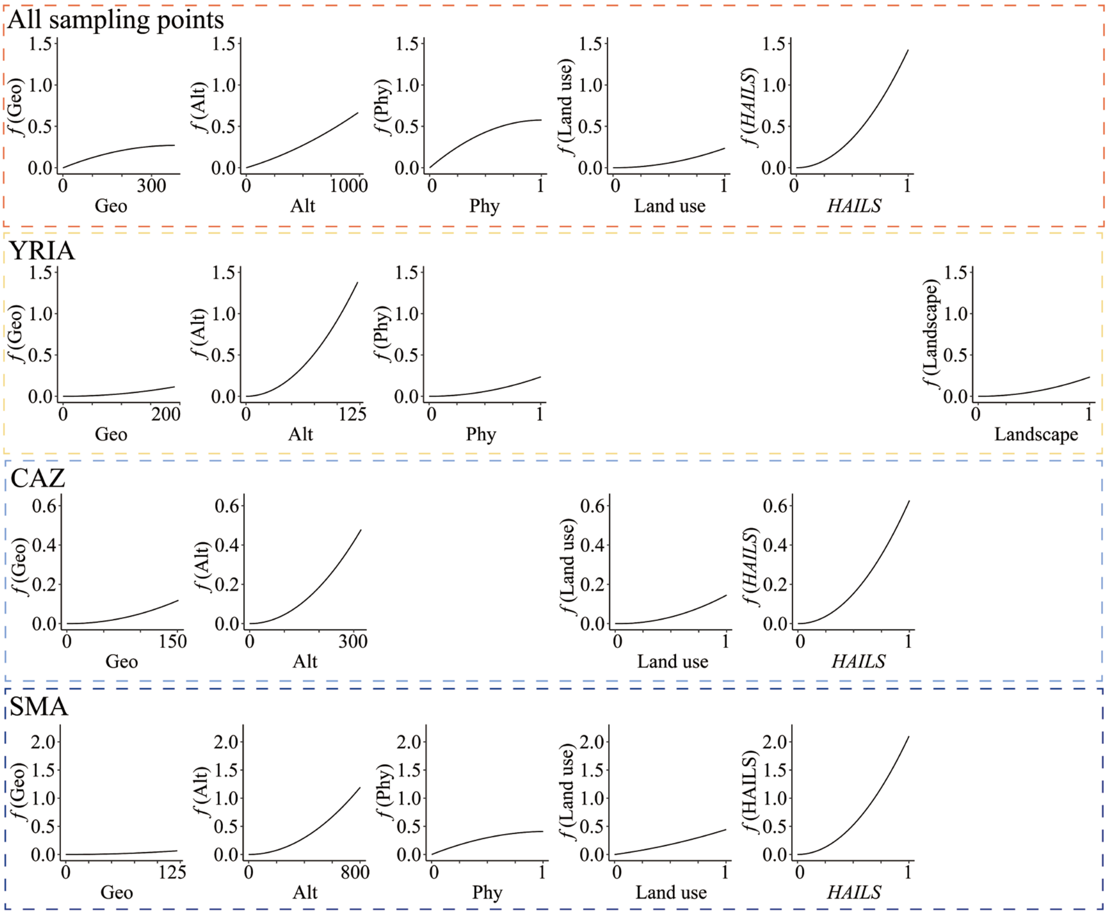


**Figure S9.** Partial response graph of GDM for analyzing $\beta_{sor}$ in October

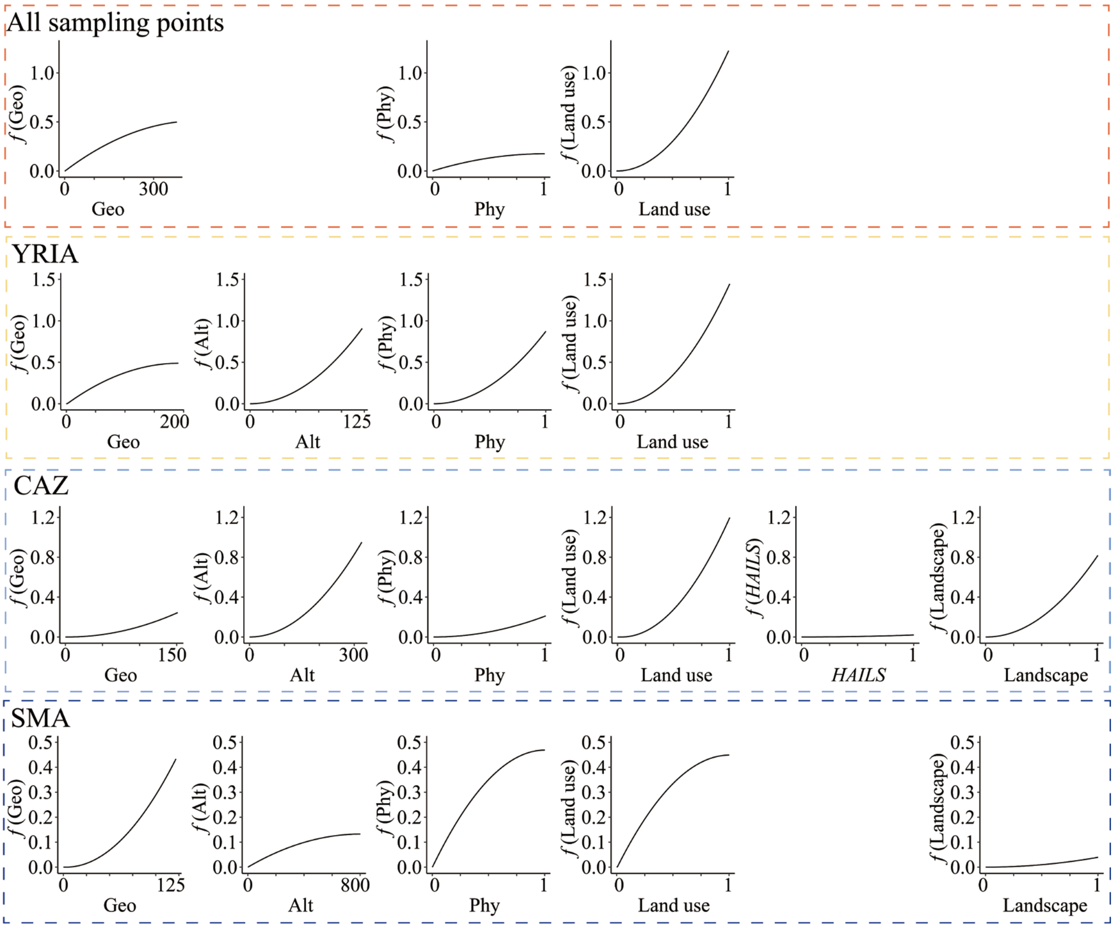


**Figure S10.** Partial response graph of GDM for analyzing $d_{BC}$ in April

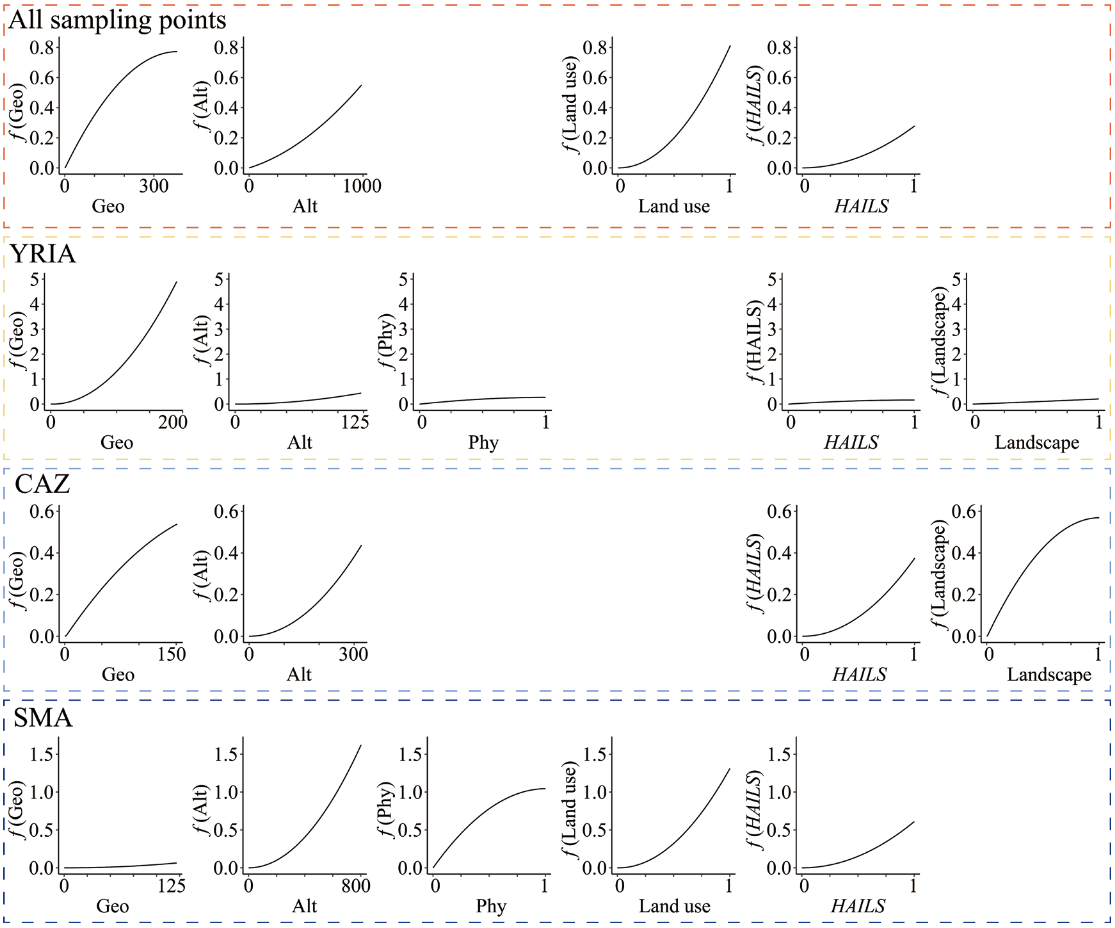


**Figure S11.** Partial response graph of GDM for analyzing $d_{BC}$ in July

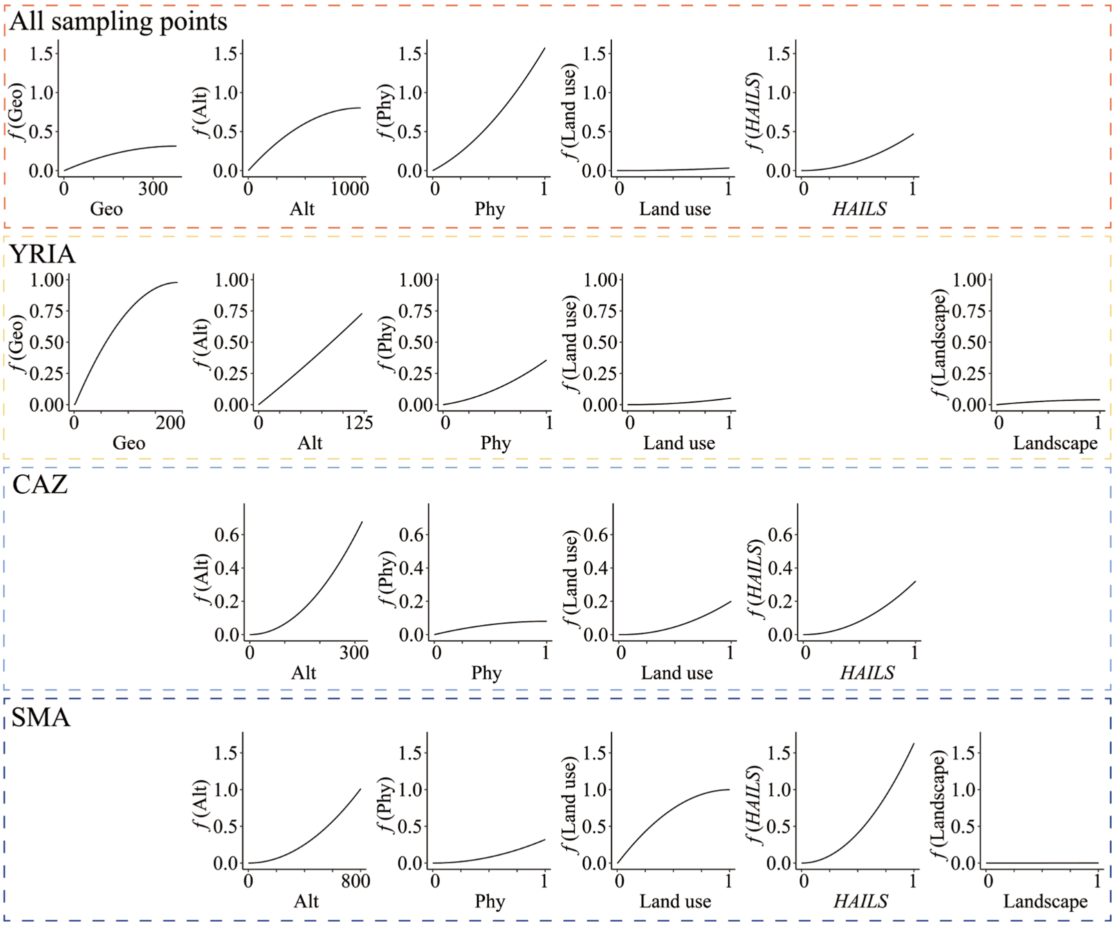


**Figure S12.** Partial response graph of GDM for analyzing $d_{BC}$ in October

## References

1. Xu, Y.; Xu, X.R.; Tang, Q. Human activity intensity of land surface: Concept, methods and application in China. *J. Geogr. Sci.* **2016**, 26(9), 1349–1361. https://doi.org/10.1007/s11442-016-1331-y.

2. Pesce, S.F.; Wunderlin, D.A. Use of water quality indices to verify the impact of Cordoba City (Argentina) on Suquia River. *Water Res*. **2000**, 34(11), 2915-2926. https://doi.org/10.1016/S0043-1354(00)00036-1.

3. Sun, W.; Xia, C.Y.; Xu, M.Y.; Guo, J.; Sun, G.P. Application of modified water quality indices as indicators to assess the spatial and temporal trends of water quality in the

Dongjiang River. *Ecol. Indic*. **2016**, 66, 306-312. https://doi.org/10.1016/j.ecolind.2016.01.054.

4. Wang, J.L.; Fu, Z.S.; Qiao, H.X.; Liu, F.X. Assessment of eutrophication and water quality in the estuarine area of Lake Wuli, Lake Taihu, China. *Sci. Total Environ*. **2019**, 650: 1392-1402. https://doi.org/10.1016/j.scitotenv.2018.09.137.
5. Wu, Z.S.; Wang, X.L.; Chen, Y.W.; Cai, Y.J.; Deng, J.C. Assessing river water quality using water quality index in Lake Taihu Basin, China. Science of the Total Environment. 2018, 612, 914-922. https://doi.org/10.1016/j.scitotenv.2017.08.293.
6. Tian, Y.L., Jiang, Y., Liu, Q., et al., 2019. Using a water quality index to assess the water quality of the upper and middle streams of the Luanhe River, northern China. *Sci. Total Environ*. **2019**, 667, 142-151. https://doi.org/10.1016/j.scitotenv.2019.02.356.
